# Coronary Arteries: Normal Anatomy With Historical Notes and Embryology of Main Stems

**DOI:** 10.3389/fcvm.2021.649855

**Published:** 2021-05-31

**Authors:** Gaetano Thiene, Carla Frescura, Massimo Padalino, Cristina Basso, Stefania Rizzo

**Affiliations:** Department of Cardiac, Thoracic, Vascular Sciences and Public Health, University of Padua Medical School, Padua, Italy

**Keywords:** embriology, anatomy, coronary arteries, history, normal variants

## Abstract

Anatomy of subepicardial coronary arteries became a topic of investigation at autopsy in Florence (Italy) by Banchi in the early twentieth century, with the discovery of dominant and balanced patterns. Thereafter, in the 60's of the same century Baroldi in Milan did post-mortem injection with spectacular three-dimensional casts. Later Sones at the Cleveland Clinic introduced selective coronary arteriography for *in vivo* visualization of coronary arteries. In the present chapter we show these patterns, as well as normal variants of origin and course with questionable risk of ischemia, like myocardial bridge as well as origin of the left circumflex coronary artery from the right sinus with retroaortic course. As far as embryology, the coronary arteries and veins are epicardial in origin and finally connect the former with the aorta, and the latter with the sinus venosus. At the time of spongy myocardium, intramural blood supply derives directly by the ventricular cavities, whereas later, at the time of myocardial compaction, vascularization originates from the subepicardial network. The connection of the subepicardial plexus with the aorta occurs with prongs of the peritruncal ring, which penetrate the facing aortic sinuses. Septation of truncus arteriosus is not responsible for the final position of the coronary orifices. Infact in transposition of the great arteries coronary ostia are regularly located within facing sinuses of the anterior aorta.

## Introduction

This chapter on anatomy and embryology of coronary arteries (CAs) has been written having in mind that the target readers are clinicians. Explaining embryology is a difficult task, and we did our best to simplify the message and facilitate the comprehension. Coronary arteries anomalies and their clinical implications are the argument of another chapter from our group for this e-book.

Moreover, it has been our deliberate intention to cover some history of CA anatomy, with relevant illustrations, to enhance the interest of the readers.

## Historical Notes

The pathology of CAs started to be a topic of interest and investigation at the beginning of the twentieth century, when myocardial infarction was found to be related to coronary obstruction. Even Morgagni in his book, dated 1761, skipped the issue (1).

**Figure 1:**
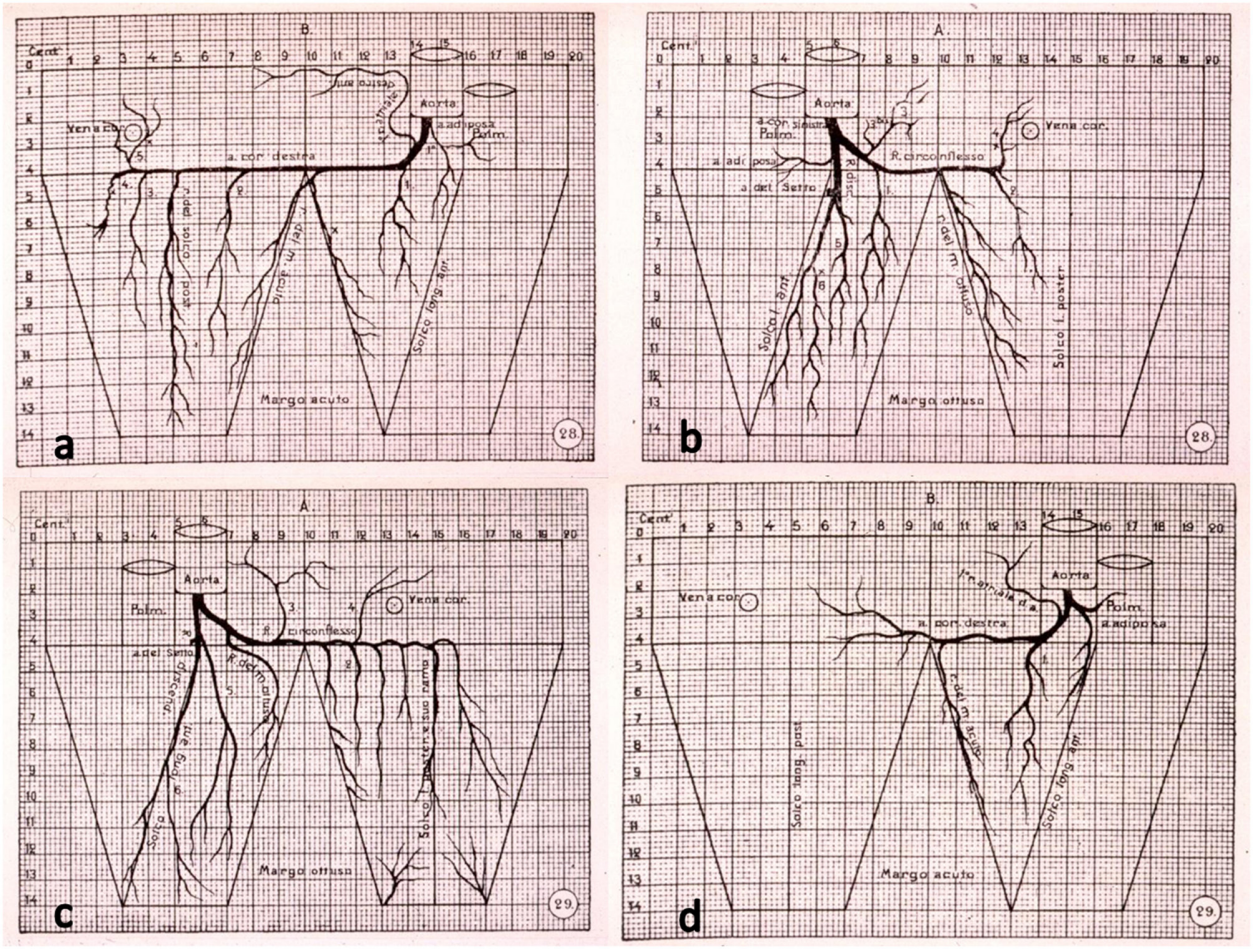
The original drawings of coronary artery dominant patterns, from Banchi (2). **(a,b)** Right dominance and **(c,d)** left dominance.

Antonio Banchi, an anatomist in Florence, in 1903 first published a milestone paper, written in Italian, on subepicardial CAs in normal hearts and introduced the concept of right and left dominance as well as balanced coronary circulation (2), according to the CA supply of the posterior wall of the left ventricle (Figure 1).

**Figure 2:**
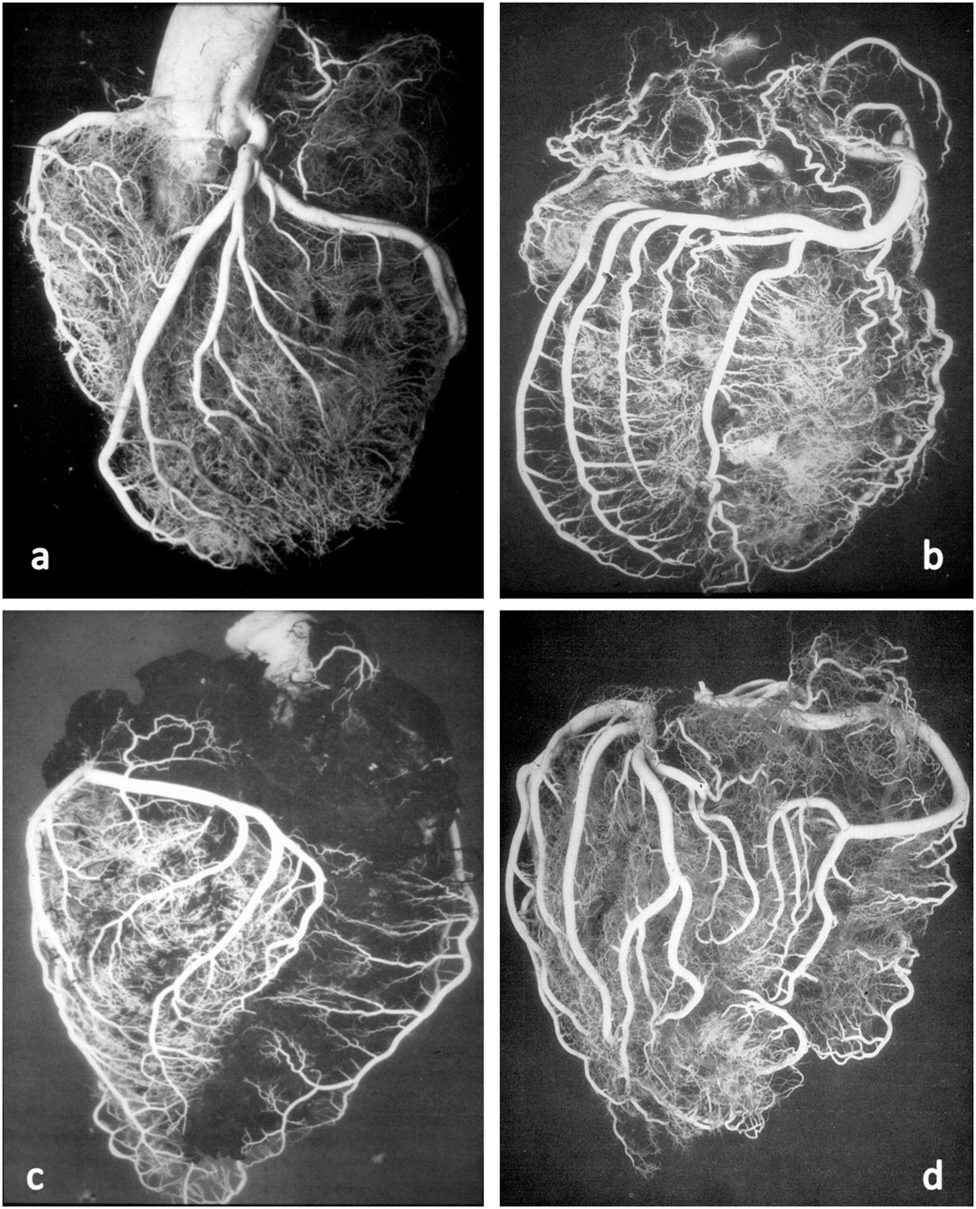
Postmortem casts of coronary arteries. From Baroldi and Scomazzoni (3). **(a)** Left coronary artery anatomy, **(b)** dominant right pattern, **(c)** dominant left pattern, and **(d)** balanced pattern.

These anatomical patterns of the coronary arterial system were confirmed through postmortem injection and casts by Giorgio Baroldi in 1963 (Figure 2) (3). The plastic substances were “Geon Latex 576” and “Neoprene 842A.” These substances, which are fluid at room temperature, solidify at 40–50°C. They were injected into the aorta under pressure, ranging from 130 to 200 mmHg, maintained for a 5- to 10-min period.

With the latex maintained under pressure, it was solidified by placing the heart in 10% formalin at 40–50°C from 48 to 72 h. Following solidification of the latex, the heart was allowed to further fix in a new 10% formalin solution at room temperature for a period of 12–48 h. Finally, the injected and fixed heart was digested in a concentrated hydrochloric acid solution.

The invention and clinical diagnostic application of selective coronary arteriography by Sones at the Cleveland Clinic (4) permitted to identify these arterial patterns *in vivo*, as a requisite for bypass surgery.

## Anatomy of Coronary Arteries

**Figure 3:**
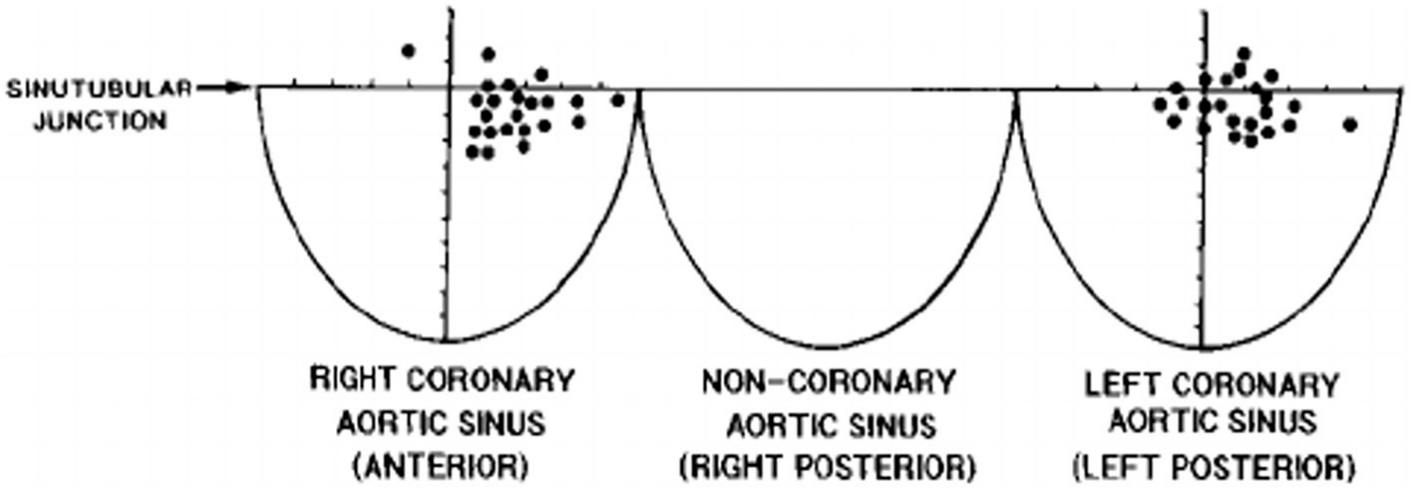
The topographical variability of coronary artery orifices in normal hearts. From Muriago et al. (5).

**Figure 4:**
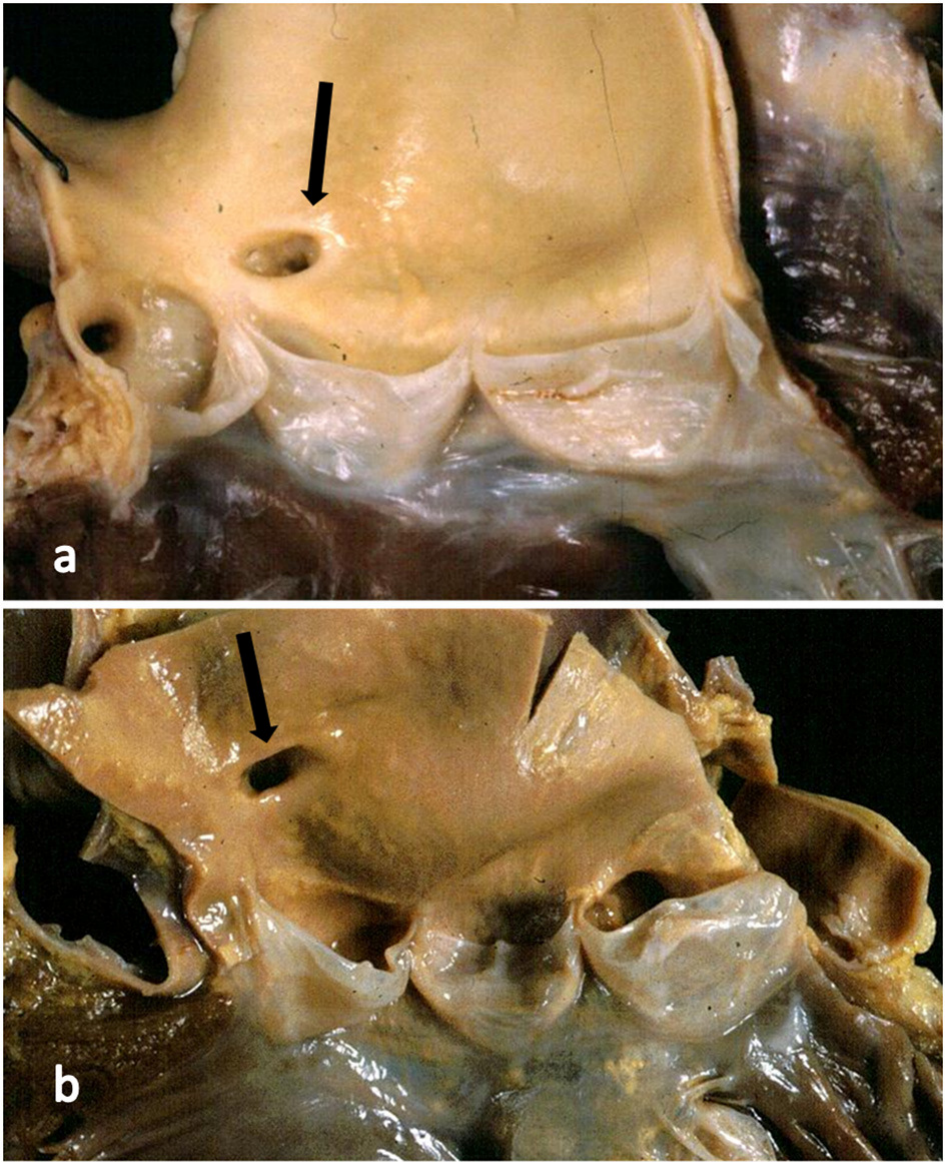
**(a)** The right coronary artery orifice (arrow) is just above (2 mm) the sinotubular junction, within normal limits. **(b)** The right coronary orifice (arrow) is well above the sinotubular junction (10 mm), over the threshold of normal limits.

**Figure 5:**
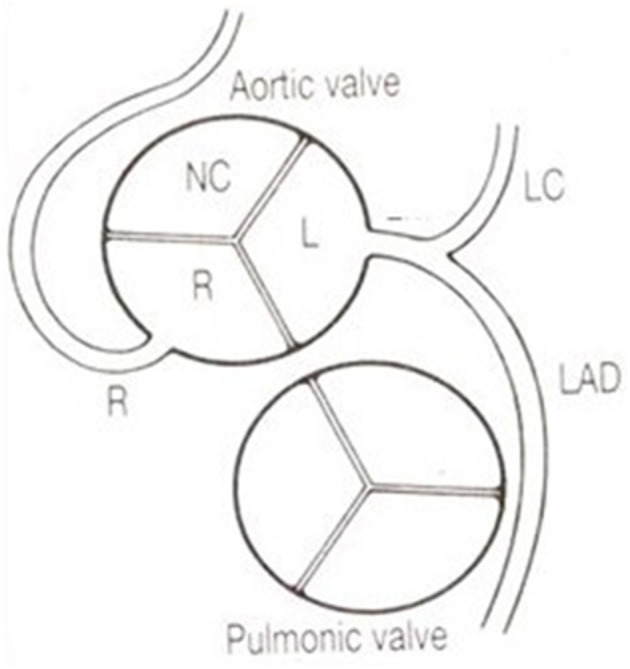
The coronary arteries arise from the facing aortic sinuses, perpendicular to the aortic wall, and the pulmonary root does not interfere with their proximal course. Note the left stem dividing into the left anterior descending (LAD) and the left circumflex (LC) branches. From Roberts (6).

**Figure 6:**
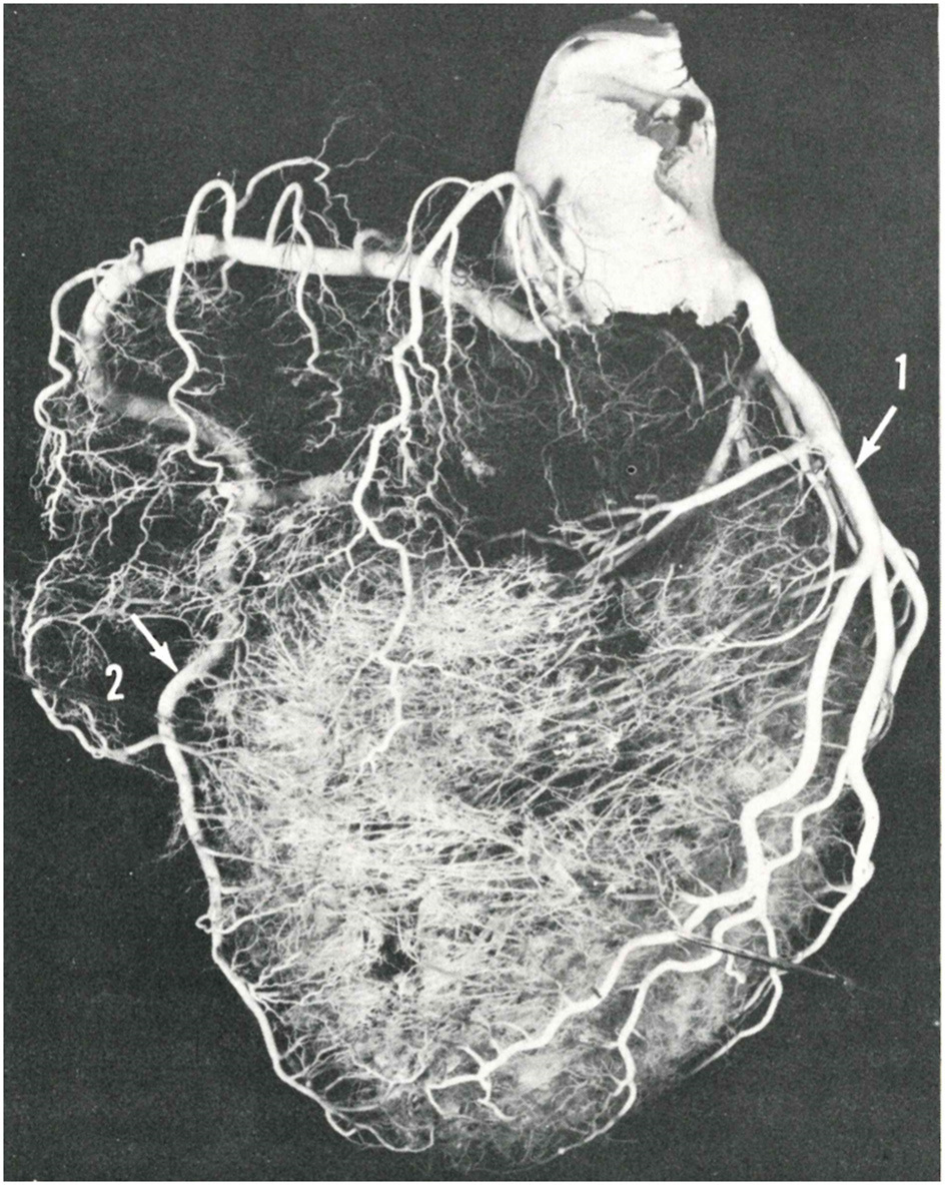
The blood flow of the ventricular septum is supported by perforating branches, originating from the anterior and posterior descending coronary arteries. From Baroldi and Scomazzoni (3).

The CAs take origin from the sinus portion of the aortic root, with the orifices located usually at the sino-tubular junction, with a variability of up to 2.5 mm (5) (Figures 3, 4). The left coronary ostium is located in the left anterior aortic sinus and the right coronary ostium in the right anterior aortic sinus, namely, the aortic sinuses facing the pulmonary artery (6) (Figure 5). Right and left CAs originate perpendicularly from the aorta, and their proximal course is not hindered by the pulmonary trunk (Figure 5). Usually, a single orifice is located in the left aortic sinus, giving origin to the left main trunk, which divides into anterior descending and left circumflex coronary branches (Figures 2a, 5). The course of the former occurs over the interventricular septum while the latter in the left atrioventricular (av) groove, with different lengths according to the dominant pattern. An intermediate artery may originate in between the two, so that the left main stem trifurcates. From the left anterior descending CA, diagonal lateral arteries take origin, for the blood supply of antero-lateral free wall of the left ventricle (Figure 2a), and perforating arteries, for the blood supply of two-thirds of the anterior ventricular septum (Figure 6).

**Figure 7:**
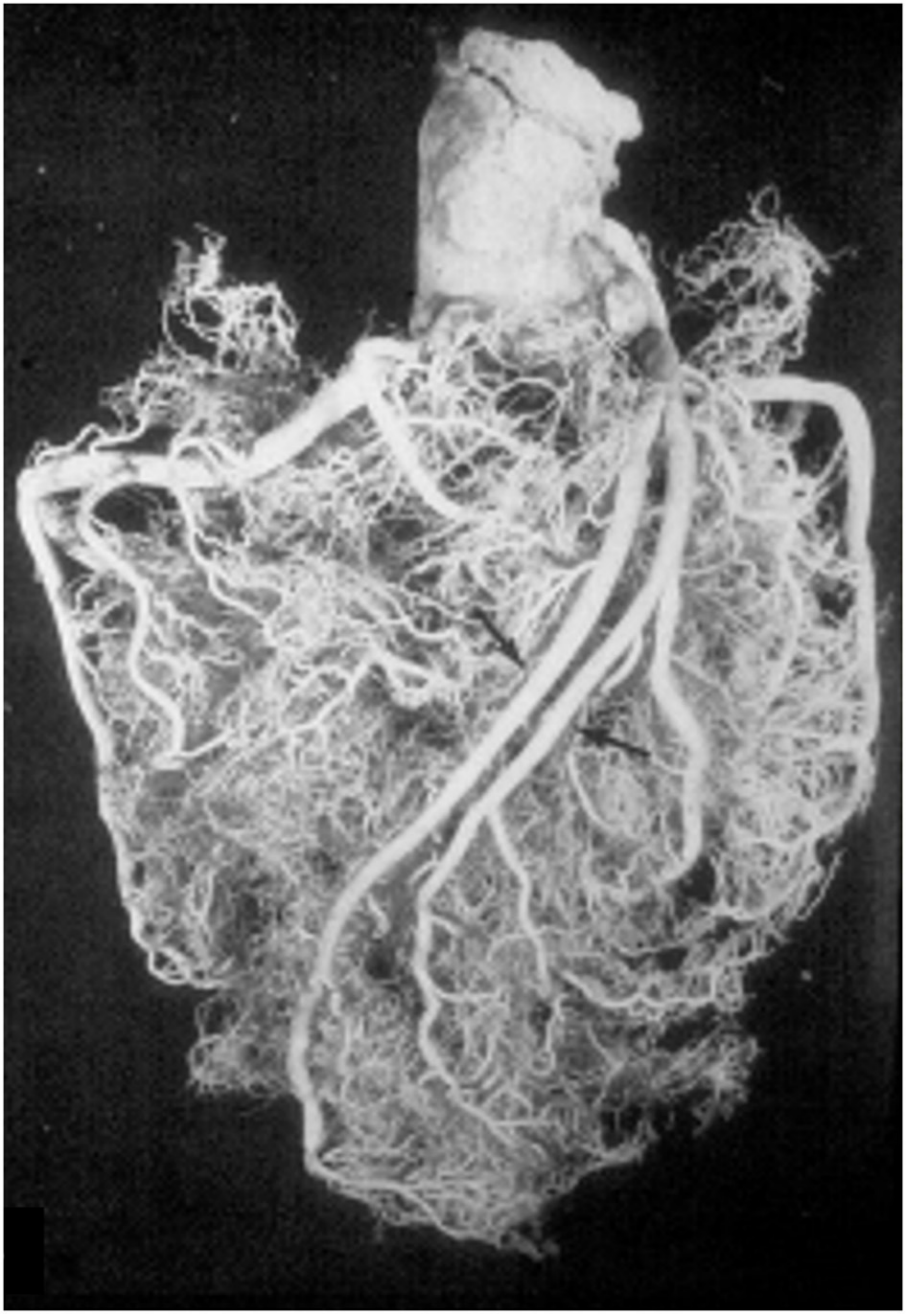
A double left anterior descending coronary artery (arrows). From Baroldi and Scomazzoni (3).

A normal variant is the double descending CA (Figure 7), the interventricular one originating septal arteries and the other one diagonal branches.

From the left circumflex artery, the obtuse angle branch takes origin to supply the left lateral wall. It may represent the end of the left circumflex artery, in case of extremely dominant right CA.

According to Baroldi (3), a right dominant pattern is present in 79% of cases, left dominant in 7% and balanced in 14%. In two-thirds of the cases, the left anterior descending CA turns at the apex and runs in the posterior interventricular groove, even up to the crux cordis.

The left circumflex artery may originate from a separate orifice of the left aortic sinus, a condition considered a normal variant since it does not imply myocardial ischemia.

The same occurs to the right CA, since a conus artery almost regularly arises from a separate, anteriorly located small orifice. The right CA runs in the right av groove and reaches the crux cordis in a right dominant pattern, giving origin to the descending CA (Figures 1a, 2b) to take over the blood supply of the posterior third of the ventricular septum. It continues in the posterior left av sulcus, originating branches for the blood supply of the posterior left ventricular free wall.

Branches for the conduction system take origin from the dominant right CA, proximally the sinoatrial node artery and distally the av node artery from the crux cordis. In the setting of left dominant CA, both of these two small arteries originate from the left circumflex artery.

**Figure 8:**
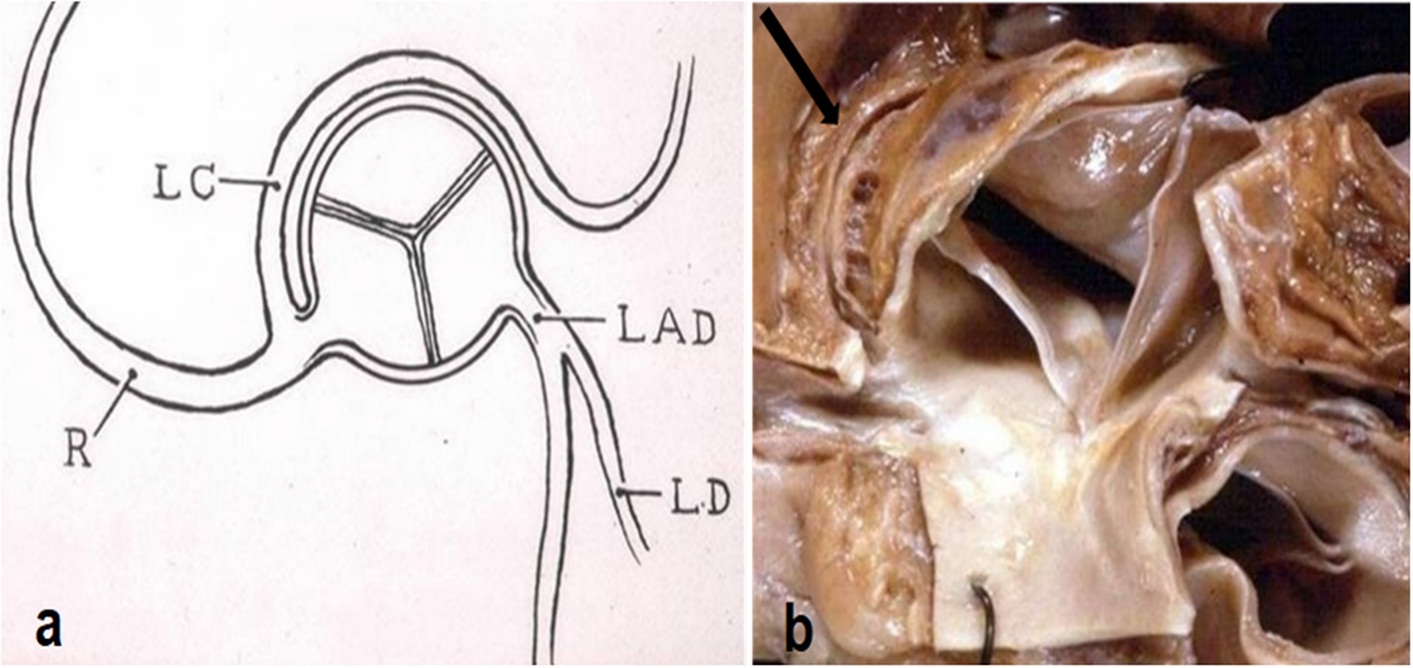
**(a)** Diagram with origin of the left circumflex artery from the right coronary artery and retroaortic course. From Roberts (6). **(b)** Gross view of the aortic root. Arrow indicates the retroaortic course of the anomalous left circumflex artery.

Another quite intriguing condition is the origin of the left circumflex artery from the right CA or from the right anterior aortic sinus itself, with a retroaortic course before reaching the left av sulcus (Figure 8). It has been observed in cases of sudden death as infarct-related artery in a postero-lateral myocardial infarction in the absence of any other explanation (7).

**Figure 9:**
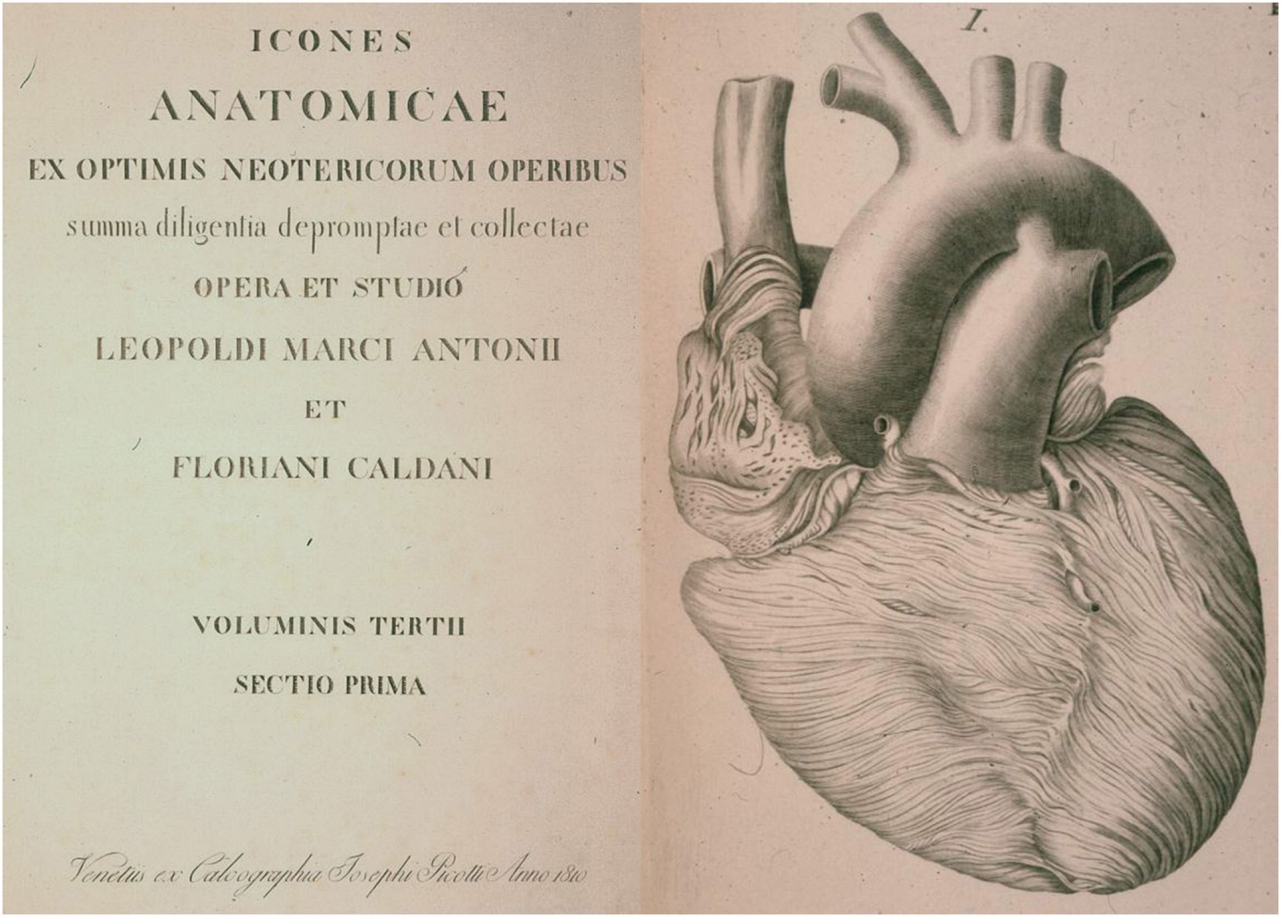
The first historical description of myocardial bridge made in 1834 by Leopoldo and Floriano Caldani, Professors of Anatomy at the University of Padua. From Caldani et al. (8).

**Figure 10:**
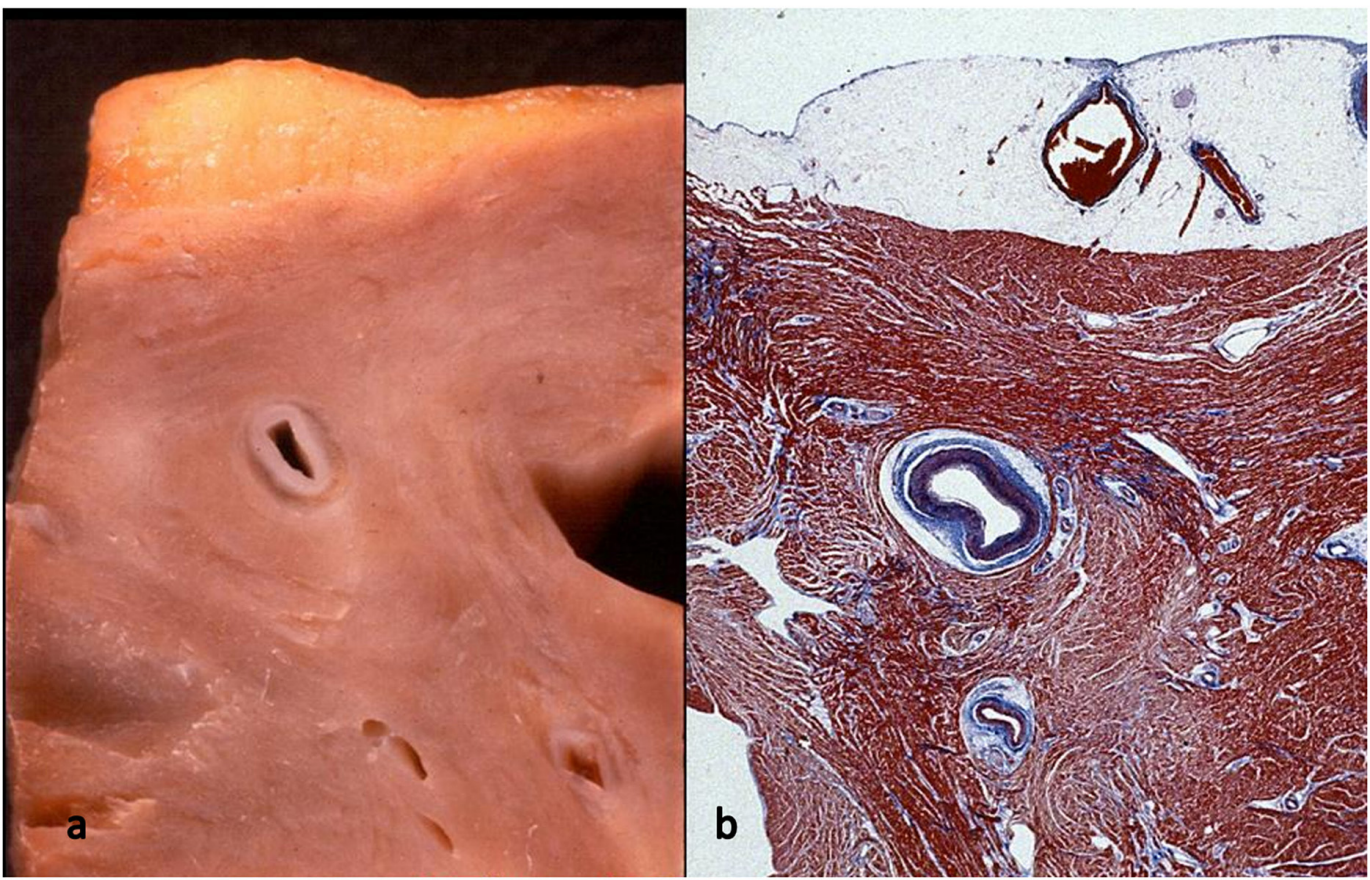
Myocardial bridge with deep intramural course: the coronary segment is completely surrounded by a myocardial sleeve. Gross **(a)** and histological views **(b)**. Azan Mallory stain.

Nearly 30% of the population shows an intramural course of the proximal left descending CA (8–11) (Figure 9). It is considered a variant of normal when just covered by a myocardial bridge. It may be a cause of myocardial ischemia if completely surrounded by a sleeve of the myocardium with disarray and an intramyocardial course at least 2.5 cm long and 0.5 cm deep (9–11) (Figure 10). The condition is particularly at risk when the intramyocardial course is just over the first septal perforating artery (Figure 6).

**Figure 11:**
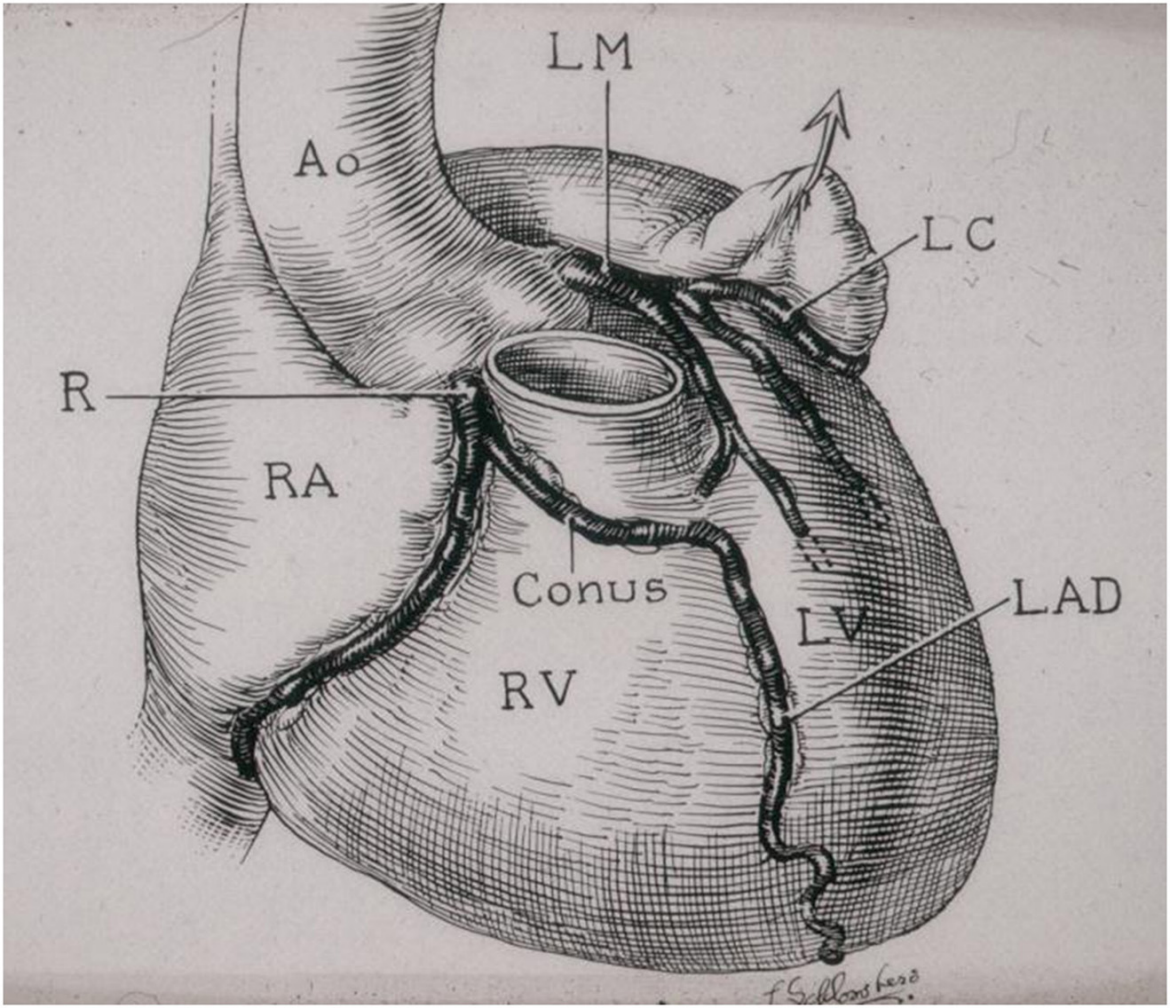
Origin of the left anterior descending coronary artery from the right coronary artery. Note the anomalous proximal course in front of the pulmonary infundibular. From Roberts (6).

The origin of the left anterior descending CA from the proximal right CA is also another normal variant, since it does not entail myocardial ischemia (Figure 11). However, the surgeon should be well aware of this unpredictable course while repairing conotruncal congenital heart diseases with a transannular patch or conduit implant.

The major subepicardial CAs are of medium size and, as such, with a “muscular” tunica media. After birth, a myointimal layer develops with time, due to migration of smooth muscle cells from the tunica media (12).

## Embryology of Coronary Stems

Both subepicardial CAs and veins are extracardiac in origin, deriving from epicardial cells (13, 14).

Their development begins with the formation of a plexus-like vasculature located in the subepicardium, which invades the myocardium and develops small vessels and capillaries.

**Figure 12:**
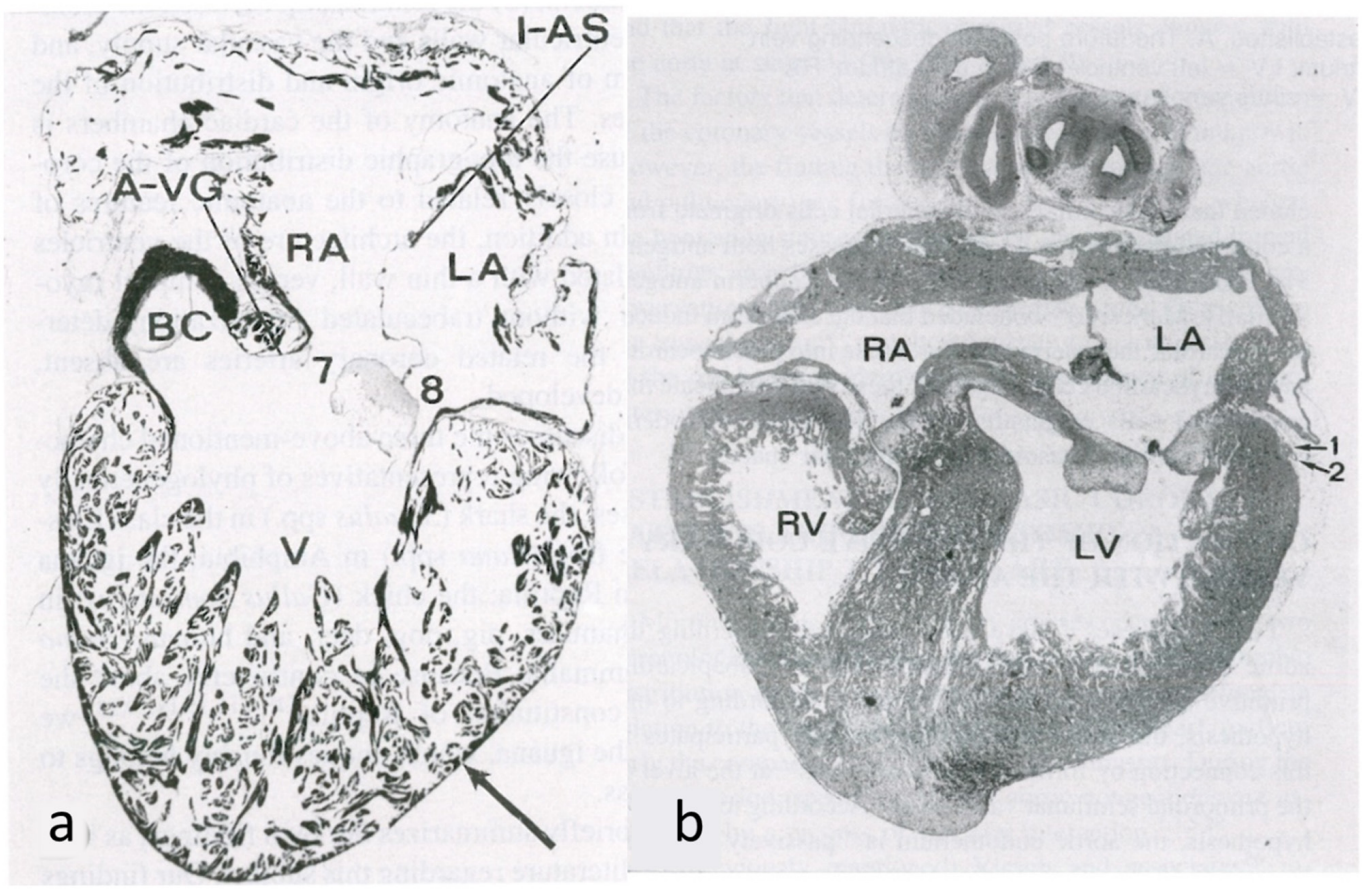
Embryology of the myocardium. **(a)** Spongy myocardium from a mature fog with blood supply deriving directly from the endocardium of the ventricular cavities. **(b)** The myocardium of a fetal chick heart becomes compact, with blood supply deriving from the subepicardial vasculature. From de la Cruz et al. (13).

Earlier, the myocardial blood supply derives directly from the ventricular cavities through the intertrabecular spaces lined by the endocardium (Figure 12a). This source of blood to the primitive spongy myocardium disappears with the myocardial compaction (Figure 12b). At this point, the whole intramyocardial vascularization consists of vessels with the endothelium derived from the subepicardium (14–16).

The origin of CAs and veins, whether intra- or extramural, is similar. Their definitive identity and function depend upon the connection, arteries with the aorta, and veins with the sinus venosus.

**Figure 13:**
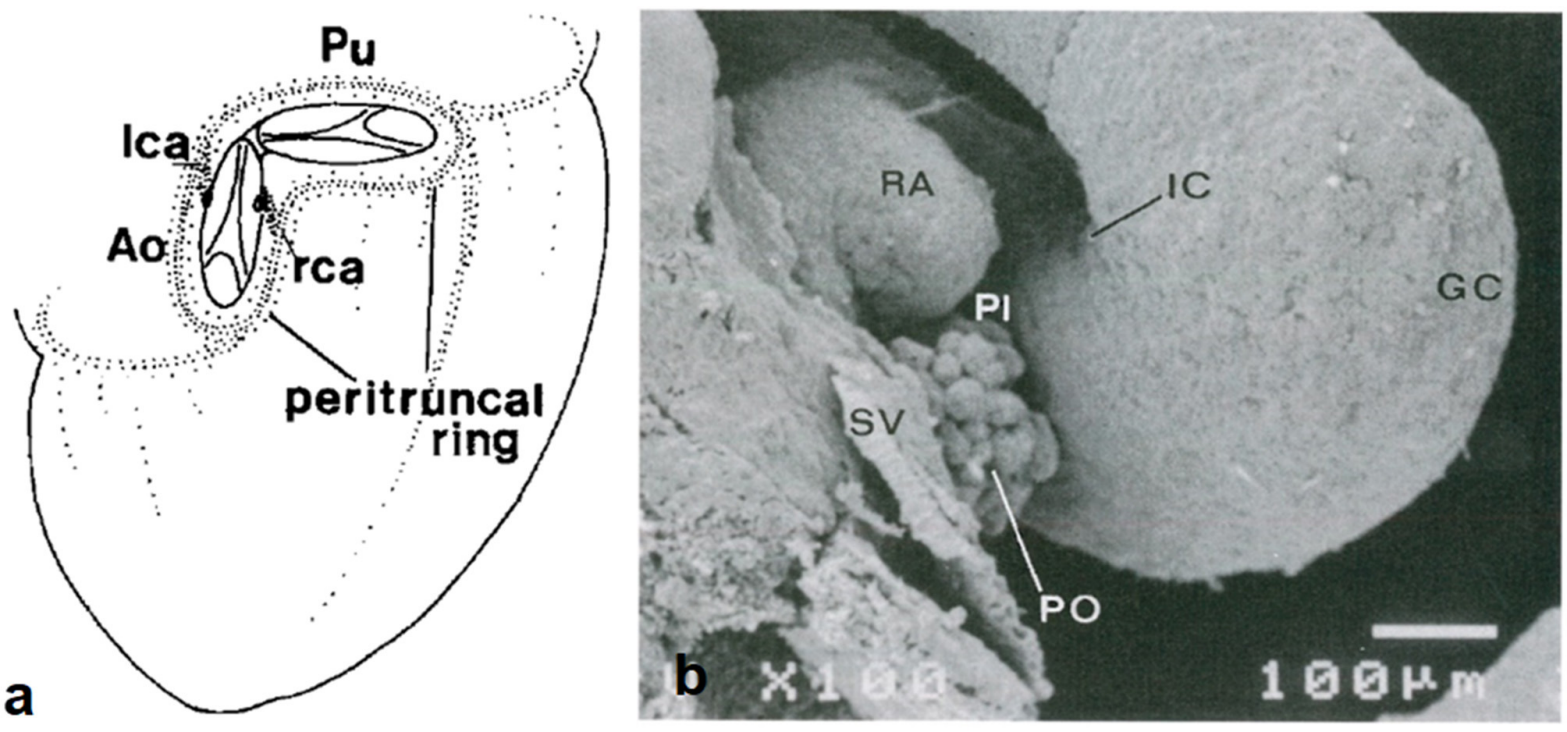
Origin of coronary arterial stems from the peritruncal epicardial ring. **(a)** From Bogers et al. (17). **(b)** From de la Cruz et al. (13).

A subepicardial network of cells (“bioepicardial organ”) surrounds the orifices of the great arteries (peritruncal ring) (Figure 13) and eventually connects with the facing aortic sinuses.

The question is whether the development of CA origin is a matter of ingrowth or outgrowth. There are two hypotheses to explain the connection.

**Figure 14:**
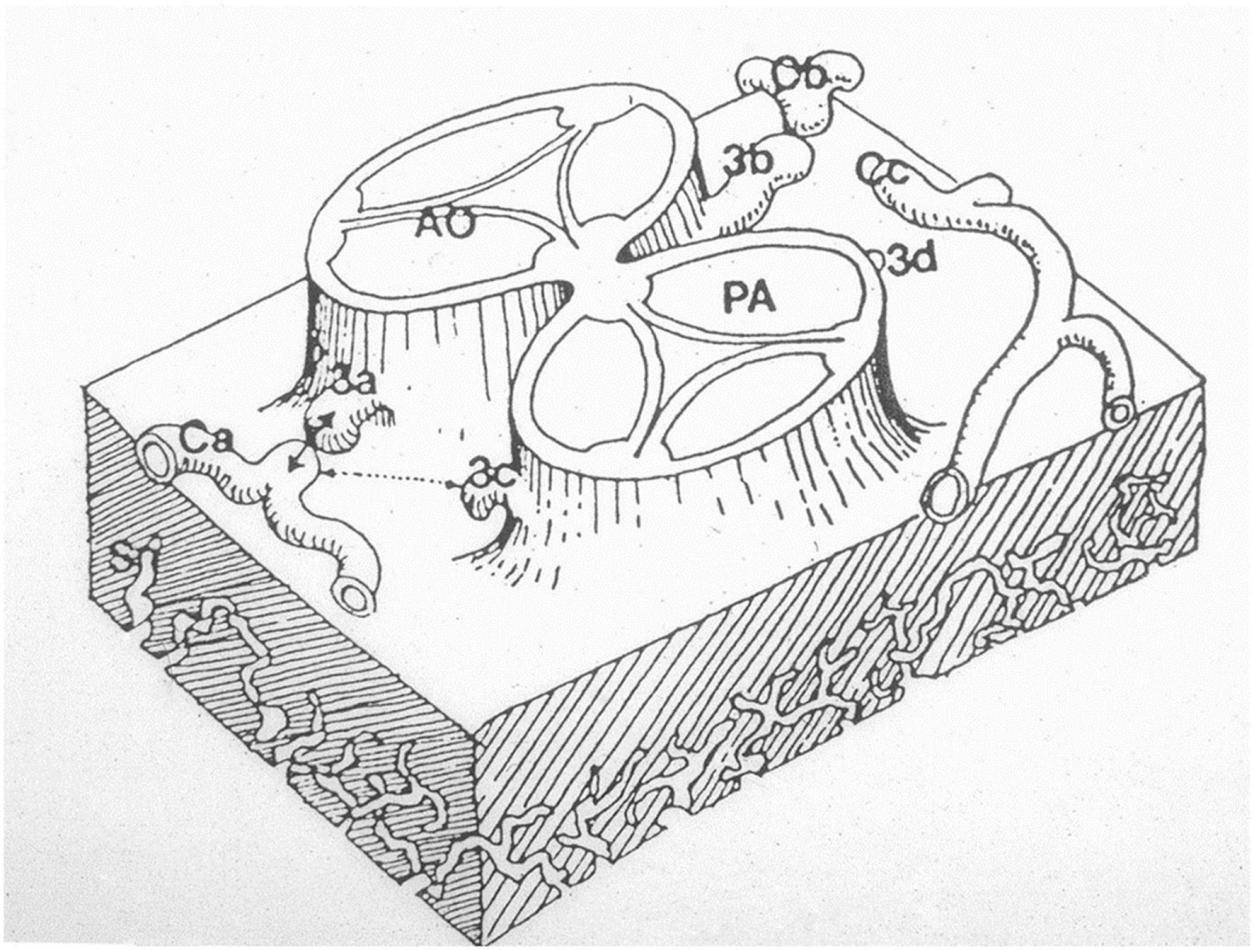
The hypothesis according to which sprouts or buds arise from the facing aortic sinuses and make contact with the subepicardial coronary vasculature. From Angelini (18).

The first is the outgrowth hypothesis, namely, the development of sprouts or buds from the aortic wall of facing sinuses, capturing the peritruncal ring of coronary subepicardial arterial vasculature (13, 18) (Figure 14).

The second developmental hypothesis, proven by serial sectioning of both human and rat embryos, is supported by the observation that the prongs of the peritruncal ring penetrate the aortic wall and make contact with the endothelial lining of the aorta (17, 19).

**Figure 15:**
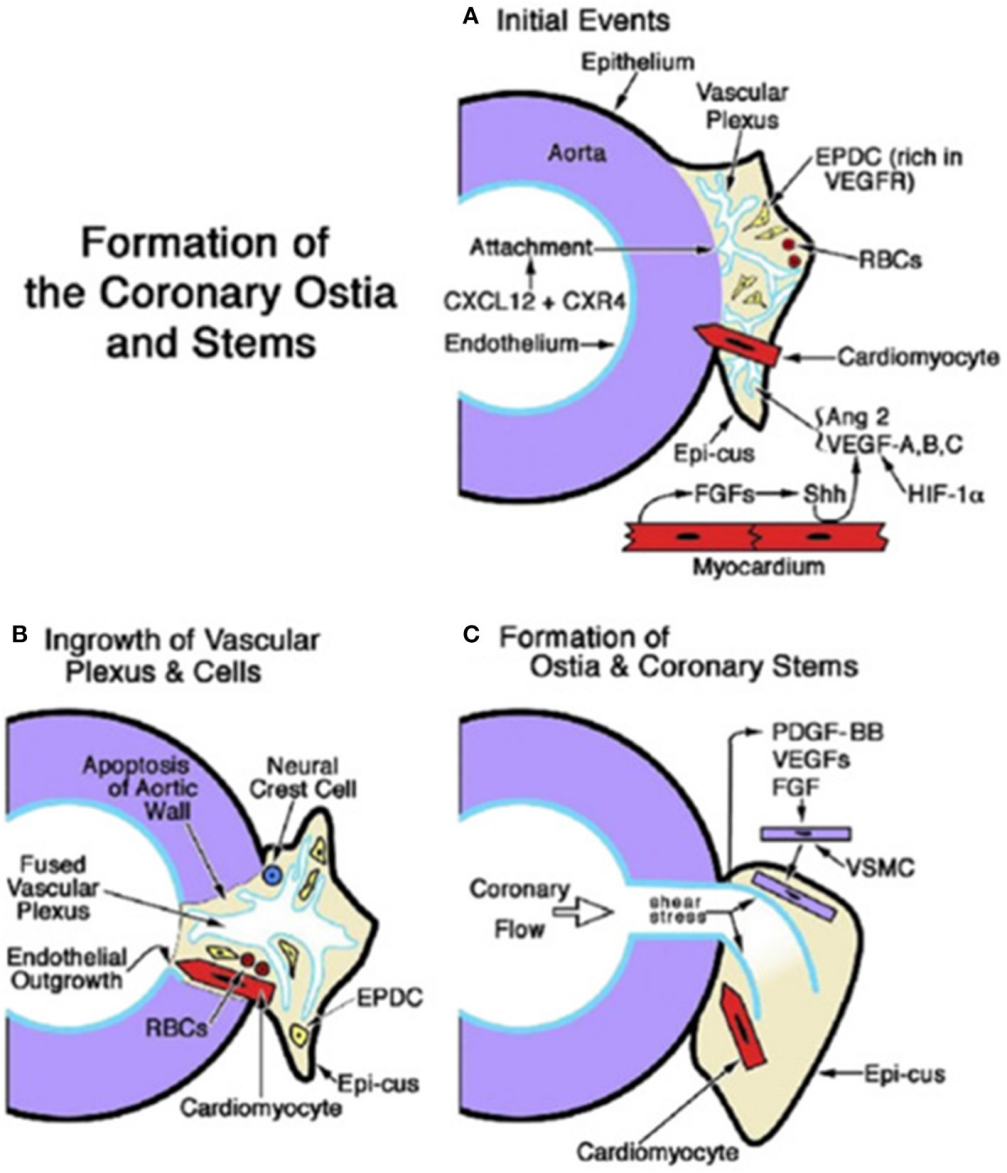
Embryology of coronary artery orifices and main stems. **(A)** Formation of the coronary ostia and stems is initiated when the capillary ring that encircles the aortic root expands and attaches as a vascular plexus (in response to CXCL12 + CXR4). The sites of ostial formation are adjacent to an epicardial cusp (Epi-cus), a thickened portion of the subepicardium that contains epicardium-derived cells (EPDCs) that are rich in Vascular endothelial growth factor (VEGF) receptors and erythroblasts [red blood cells (RBCs)]. Cardiomyocytes guide the attachment of the vascular plexus. Fibroblast growth factors (FGFs) from the myocardium promote Sonic hedgehog (Shh) signaling, which, together with hypoxia inducible factor-1 (Hif-1), stimulates VEGFs and angiopoietin (Ang) 2, thus facilitating angiogenesis of the vascular plexus. **(B)** Myocardium-derived cardiomyocytes and neural crest cells facilitate the entry of the vascular plexus into an opening in the aortic wall, created by apoptosis. An endothelial ingrowth demarcates the pathway of the ostium formation. **(C)** The onset of coronary flow and shear stress is key to the remodeling of the vascular plexus. Differentiation, migration, and attachment of vascular smooth muscle cells (VSMCs) are influenced by (1) platelet-derived growth factor (PDGF)-BB activation in endothelial cells and the ligand's interaction with PDGFR-β in VSMC progenitors; and (2) the influence of VEGFs and fibroblast growth factors (FGFs). From Tomanek and Angelini (20).

Recent investigations (20) confirmed that the proximal CAs do not grow from the aorta; on the contrary, they grow into the aorta from the peritruncal ring of the subepicardial vascular plexus (Figure 15).

A consensus document from the Development, Anatomy, and Pathology Working Group of the European Society of Cardiology (21) was in favor of the ingrowth hypothesis with a statement that deserves full quotation “…CAs were originally thought to form by angiogenesis from the aortic root endothelium” (22). “Angiogenesis implies the formations of new vessels from pre-existing ones, mostly by means of controlled endothelial sprouting” (23). “Indeed, until the late 1980s, it was thought that CAs entirely derived from an aortic endothelial outgrowth that would expand to form the complete coronary system, including coronary veins. Further research in avian models (19, 24–26) partially argued against, demonstrating that: (i) prospective CA endothelial cells do not bud from the aortic root, but instead grow into the aortic wall from an aortic peritruncal plexus to connect to the systemic circulation, most likely under the guidance of vascular endothelial growth factor (VEGF-C) and periaortic cardiomyocytes (27) and (ii) at least part of the early arterial coronary vascular system forms through a process of vasculogenesis…and subsequent fusion of endothelial cell clusters to form new blood vessels” (28).

As CA stems connect the aorta to the ventricular coronary tree, Thevenieu-Ruissy et al. did an investigation in both wild-type and Tbx1 null mice embryos (29). They demonstrated that a periarterial plexus bridges limited outgrowth of the aortic endothelium with the ventricular plexus during CA stem development, supporting the hypothesis that outgrowth of aortic endothelium contributes little to proximal CA stems.

Septation of the arterial pole of the heart (42 days in the human embryo) precedes the appearance of coronary ostia when cells from the peritruncal ring migrate into the aortic root. Septation therefore cannot be responsible for the final position of coronary orifices.

Formation of the left CA precedes the right CA. Moreover, different from the tunica media of the ascending aorta, the tunica media of the CAs does not derive from the neural crest.

Cellular cross talks and signaling pathways (notch and hippo signals, transcription factors, angiogenic molecules, and apoptosis) take place (30–34). VEGF plays a crucial role in the development of coronary ostia and main stem formation (20) (Figure 15). Absence of VEGF was shown to inhibit ostia formation. Epicardial inhibition, reducing apoptotic remodeling at the ventricular–arterial junction, alters vascular connection with the aorta and may produce CA anomalies equal to those observed in humans (35).

Why the primitive subepicardial coronary arterial vasculature tends to connect with the facing aortic sinuses, instead with facing pulmonary sinuses, is still unknown. The explanation cannot be the posterior position of the aorta since in transposition of the great arteries (TGA), where the aorta is anterior, the CA regularly connects with the facing sinuses of the aorta. An anomalous origin of a coronary artery from the posterior pulmonary artery in TGA is indeed extremely rare (36, 37).

## Author Contributions

GT designed and drafted the work. CF, MP, CB, and SR revised the work. All authors contributed to the article and approved the submitted version.

## Conflict of Interest

The authors declare that the research was conducted in the absence of any commercial or financial relationships that could be construed as a potential conflict of interest.
